# Allele mining of eukaryotic translation initiation factor genes in *Prunus* for the identification of new sources of resistance to sharka

**DOI:** 10.1038/s41598-023-42215-w

**Published:** 2023-09-14

**Authors:** David Tricon, Julie Faivre d’Arcier, Jean-Philippe Eyquard, Shuo Liu, Stéphane Decroocq, Aurélie Chague, Weisheng Liu, Gulnara Balakishiyeva, Alamdar Mammadov, Timur Turdiev, Tatiana Kostritsyna, Bayram M. Asma, Zeynal Akparov, Véronique Decroocq

**Affiliations:** 1 INRAE, UMR 1332 BFP, Virologie, 71 Avenue Edouard Bourlaux, 33882 Villenave d’Ornon, France; 2 UMR 1332 BFP, CS20032, Université de Bordeaux, 33882 Villenave d’Ornon, France; 3 Liaoning Institute of Pomology, Tiedong Street, Xiongyue Town, Bayuquan District, Yingkou, 115009 Liaoning China; 4 Institute of Molecular Biology and Biotechnologies, Ministry of Science and Education, 11 Izzat Nabiev Str., 1073 Baku, Azerbaijan; 5 Institute of Plant Biology and Biotechnology, Timiryazev Str. 45, 050040 Almaty, Kazakhstan; 6 International Higher School of Medicine, 1F Intergelpo Street, 720054 Bishkek, Kyrgyzstan; 7 Department of Horticulture, Malatya Turgut Ozal University, Malatya, 44210 Turkey; 8 Genetic Resources Institute of ANAS, Azadlig Ave. 155, 1106 Baku, Azerbaijan; 9 Present Address: INRAE Unité de Recherches 1052 GAFL, 67 allee des Chênes, 84143 Montfavet, France; 10 Present Address: INRAE Unité Expérimentale Domaine des Jarres, 33210 Toulenne, France

**Keywords:** Plant breeding, Biodiversity

## Abstract

Members of the eukaryotic translation initiation complex are co-opted in viral infection, leading to susceptibility in many crop species, including stone fruit trees (*Prunus* spp.). Therefore, modification of one of those eukaryotic translation initiation factors or changes in their gene expression may result in resistance. We searched the crop and wild *Prunus* germplasm from the Armeniaca and Amygdalus taxonomic sections for allelic variants in the *eIF4E* and *eIFiso4E* genes, to identify alleles potentially linked to resistance to *Plum pox virus* (PPV). Over one thousand stone fruit accessions (1397) were screened for variation in eIF4E and eIFiso4E transcript sequences which are in single copy within the diploid *Prunus* genome. We identified new alleles for both genes differing from haplotypes associated with PPV susceptible accessions. Overall, analyses showed that *eIFiso4E* is genetically more constrained since it displayed less polymorphism than *eIF4E*. We also demonstrated more variations at both loci in the related wild species than in crop species. As the eIFiso4E translation initiation factor was identified as indispensable for PPV infection, a selection of ten different eIFiso4E haplotypes along 13 accessions were tested by infection with PPV and eight of them displayed a range of reduced susceptibility to resistance, indicating new potential sources of resistance to sharka.

## Introduction

Viral diseases represent an increasing problem in modern, intensive agriculture, and the situation is expected to intensify with global warming. Control measures against viral diseases include the use of virus-free seeds or rootstocks, chemical controls of virus-transmitting vectors, and deployment of virus-resistant cultivars based on dominant or recessive resistance mechanisms^1^. While dominant resistance is, in general, an induced and race-specific resistance, recessive resistance in plant-virus interactions is more likely to derive from a passive mechanism due to the absence or to the inappropriate nature of a host factor specifically required by the virus to complete its life cycle^2^. The corresponding dominant allele, also called susceptibility allele or *(S)*-gene, is conceptually envisioned as encoding a susceptibility factor needed by the virus (see review^3^). Up to now, resistant cultivars were based, in many cases, on dominant resistance *(R)*-genes which are an attractive option for breeders because they are easy to manipulate in breeding programs. However, they are not always available in the natural diversity of crop species and their interspecific transfer from model plants to crop species proves difficult^4^. An alternative strategy is to employ recessive resistance based on the defect of a *(S)*-gene. Recent studies showed that this resistance mechanism is more easily transferred from model plants to crop species. Indeed, *(S)*-genes are constitutive host cell factors that are co-opted and required by the pathogen to complete or sustain its infectious cycle (*ex.* the translation initiation factors in^5^). They are thus expected to be highly conserved across plant genera and if a virus recruits them in a model plant, it likely uses them in its natural host crop species. This is the case of the eukaryotic translation initiation factors, eIF4E and its isoform, eIFiso4E (see review^6^). In consequence, the search for allelic variants of these genes that no longer exhibit a susceptible response, i.e., the type of host variant encountered in a compatible host/virus interaction, could potentially lead to new sources of resistance. This was demonstrated in various crop species such as tomato, melon and pepper. Allele mining by targeting *(S)*-genes in those crop species and natural populations has emerged as an important approach for cloning and characterizing new forms of disease resistance factors^7–11^.

In stone fruit tree species, sharka is the most detrimental disease, with significant socio-economic impact, especially in Europe^12^. The causative agent is a potyvirus of the species *Plum pox virus* (PPV)^13^. Few sources of resistance to this disease have been described but none of them in peach or diploid plum. Resistance to sharka was identified and documented in wild *Prunus armeniaca* (apricot)^14^ as well as in peach related species, such as *P. davidiana*^15^ and *P. dulcis* (almond)^16, 17^. Although significant effort was devoted to finding genes controlling resistance to PPV, their characterization and utilization have proven to be a long and arduous endeavor^18^. Moreover, those sources of resistance are rather limited, with one single origin per species^19^. Recent history has demonstrated the dangers of relying too heavily on such a limited resistant germplasm, especially when confronted with the diversity of the virus^13^. To diversify those sources of resistance to PPV and find new ones, other resistance mechanisms were identified in the model plant *Arabidopsis thaliana* that are linked/bound to factors of the translation initiation machinery, eIFiso4E and eIFiso4G1^20, 21^.

In eukaryotes, translation initiation factors are encoded by a small multi-gene family in which isoforms partly act redundantly. In plants, potyviruses have a specific requirement for a given protein, eIF4E/eIF4G or their isoforms eiFiso4E/eIFiso4G that depends on the host plant and on the virus^22^. For example, viruses of the species *Lettuce mosaic virus* (LMV) use eIF4E to infect *Lactuca sativa* (lettuce) but use eIFiso4E in the case of *Arabidopsis thaliana*^23^. Previous studies showed that, in the case of PPV, the eIFiso4E factor is indispensable to viral infection both in *Arabidopsis thaliana* and the European hexaploid plum *P. domestica*^20, 24^. However, in the diploid plum *P. salicina*, an RNAi silenced *eIFiso4E* transgenic plant could not be obtained by 35S-overexpressing an intron-spliced-hairpin *eIFiso4E* construct^25^. As *eIFiso4E* is a single-copy gene on the *Prunus* diploid genome, this is probably due to a lethal counter-effect of the eIFiso4E null allele on plant growth in diploid *Prunus* species. On the contrary, the silencing of one of the two copies of *eIFiso4G* displays durable and stable resistance to PPV, with no consequence onto plum tree growth^25^.

Here, our goal was to investigate natural allelic variation in the *Prunus eIF4E* and *eIFiso4E* genes among the stone fruit cultivated germplasm (apricot, almond and peach crop species) as well as their wild related, ornamental and undomesticated species. Due to mRNA length constraints, we focused in this study on the *eIF4E* and isoform *eIFiso4E* genes (600 to 700 bp long, Table 1) and not on the *eIF4G* genes (> 2.5 kb). Our first objective was to evaluate and compare *eIF4E* and *eIFiso4E* genetic diversity within the cultivated and the wild *Prunus* germplasm, according to their species and to their regions of origin. Several haplotypes with various sites comprising amino acid substitutions, insertions and deletions with however no frame-shift or stop-codon, were identified in both *eIF4E* and *eIFiso4E* sequences. Because most of the crop species of *Prunus* are susceptible to sharka, we examined intraspecific relative to interspecific gene variability to test the hypothesis that more new alleles could be found in wild related, ornamental and undomesticated species. We secondly identified haplotypes of the *eIFiso4E* with variations in the coding sequence that could confer resistance to sharka. We assessed susceptibility to PPV for rare allelic variation of the eIFiso4E susceptible factor found in less than 5% of the sequences and identified new potential sources of resistance to sharka.

**Table 1:** Number of accessions from which eIF4E and eIFiso4E sequences were retrieved.

	Number of individuals		Coding sequence length (bp)		Amino acids length	
	*eIF4E*	*eIFiso4E*	*eIF4E*	*eIFiso4E*	*eIF4E*	*eIFiso4E*
Armeniaca main group	695	767	702	639-642-645	234	213-214-215
Apricot crop species	400	413	702	639-642-645	234	213-214-215
Wild apricots	220	268	702	639-642	234	213-214
Apricot related species	75	86	702	642	234	214
Amygdalus main group	189	182	702	642-645-648	234	214-215-216
Almond crop species	134	127	702	642-645-648	234	214-215-216
Almond related species	55	55	702	642-645-648	234	214-215-216
Persica main group	284	282	702	642-645-648	234	214-215-216
Peach crop species	252	248	702	642-645-648	234	214-215-216
Peach related species	32	34	702	642-645	234	214-215

Length variations of sequences are indicated in base pairs (bp) and amino acids.

## Results

**Figure 1:**
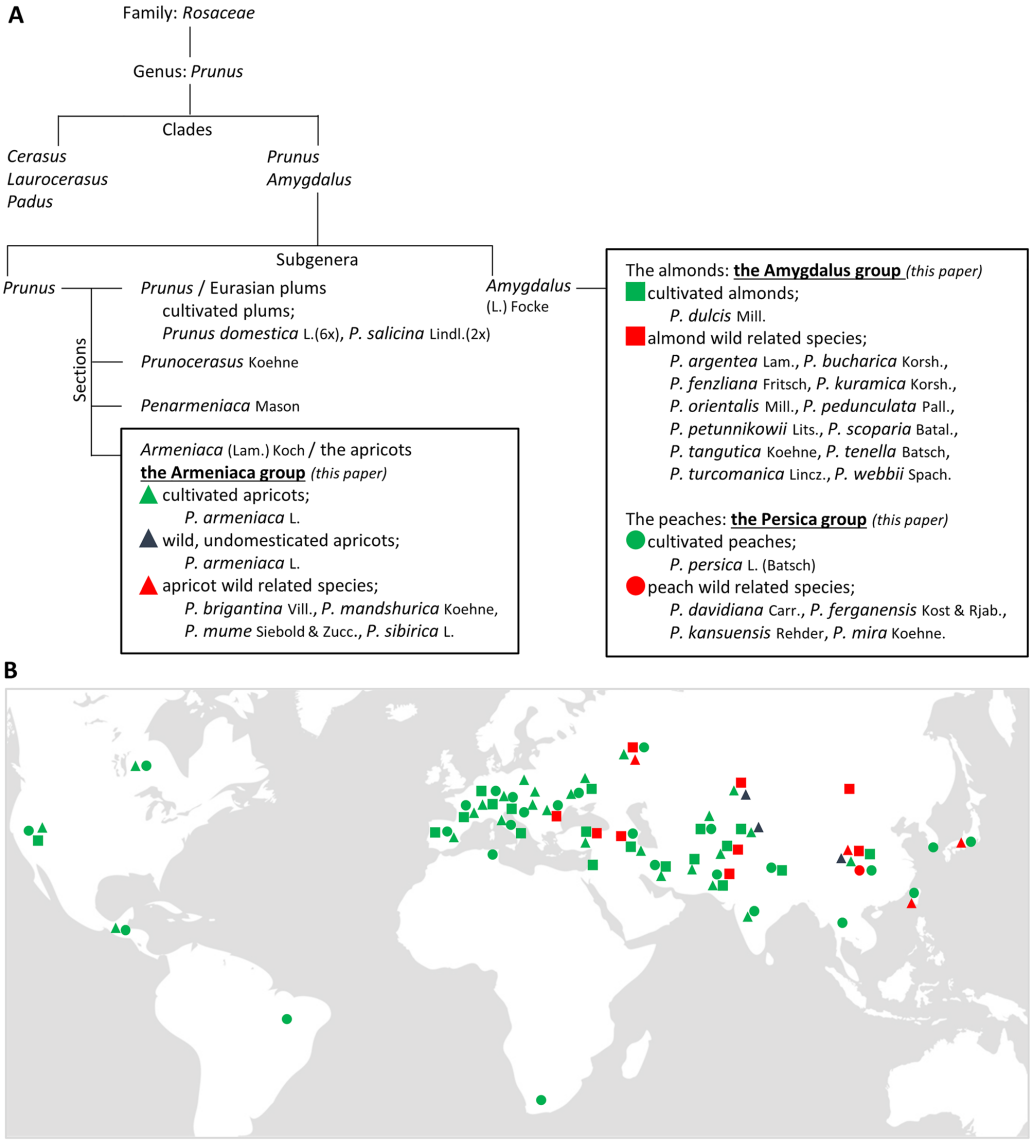
Taxonomy and geographic distribution of *Prunus* accessions. **(A**) Taxonomy and schematic phylogeny of the accessions used in this study. Classification follows previous classifications of Bortiri et al.^45^ linked with Rehder^46^ and Mason^47^. Black squares localize the three main groups of this work in the *Prunus* genus: the Armeniaca, the Amygdalus and the Persica groups representing accessions related to apricots, almonds and peaches identified by triangles, squares and circles respectively. Green, red and blue colors refer to crop species, wild related species and wild/undomesticated apricots respectively. (**B**) Geographic distribution of the accessions used in this study according to their country of initial sampling (see Table S1). The world map can be downloaded under free license at https://www.vecteezy.com/vector-art/10961532-world-map-vector-illustration-isolated-on-grey-background-flat-earth-globe-or-world-map.

Overall descriptions of the plant material of this study are reported in Fig. 1A,B and Table S1 (see below the “Plant material and sampling” section in “Materials and methods” for more details).

### *eIF4E* and *eIFiso4E* coding and amino acid sequence lengths

The *eIF4E* coding sequence was 702 base pairs long, from the start codon to the stop codon and the predicted protein was 234 amino acids long for all the accessions. Sequence length variations were observed for *eIFiso4E* due to the presence or the absence of triplets of nucleotides causing no frame shift. In consequence, the *eIFiso4E* coding sequence varies from 639 to 648 base pairs and the protein, from 213 to 216 amino acids (Table 1).

### Polymorphism and heterozygosity among the *eIF4E* and *eIFiso4E* loci

**Figure 2:**
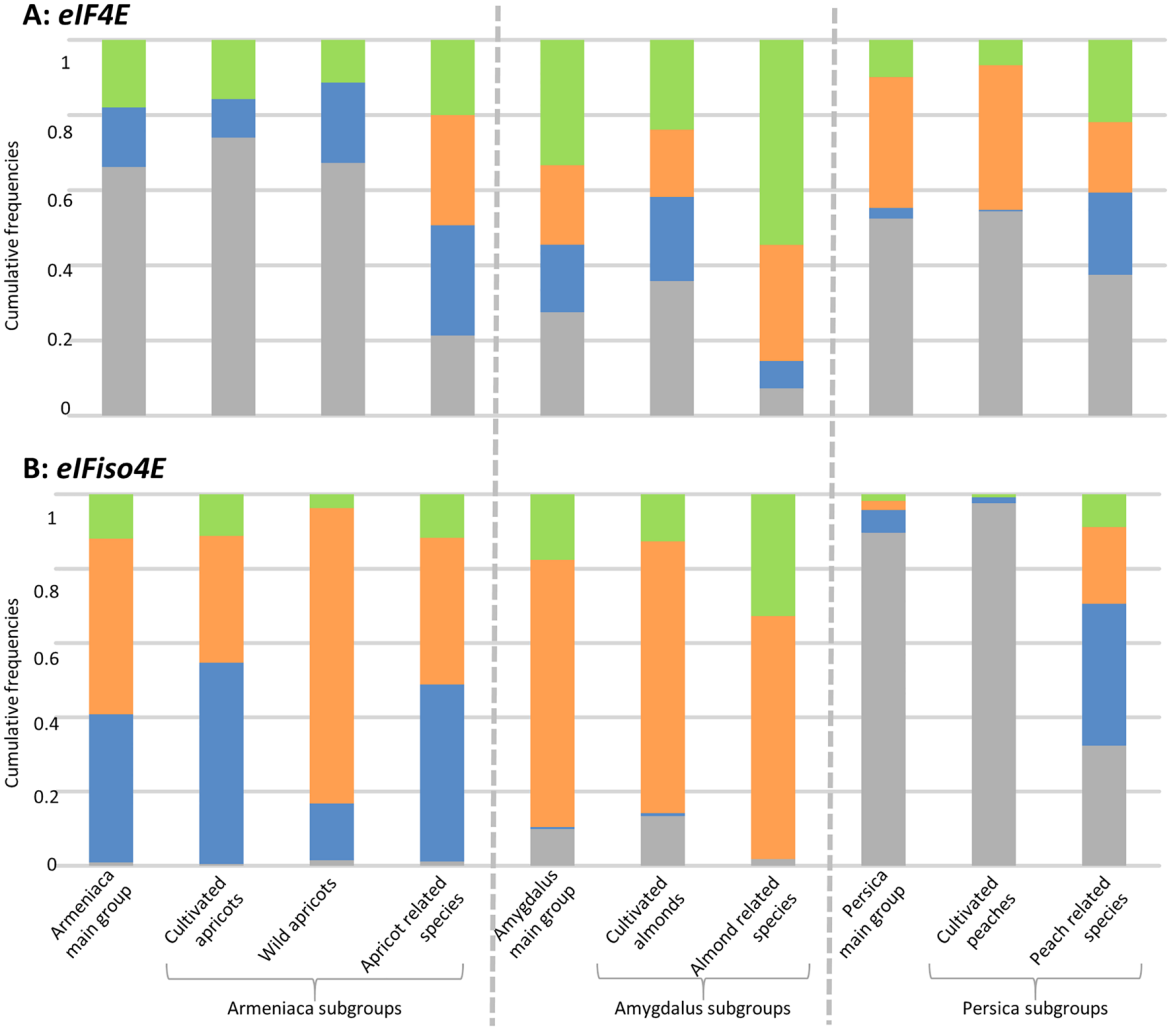
Stacked diagrams with cumulative frequencies of variations in eIF4E (**A**) and eIFiso4E (**B**) amino acid sequences within main groups and subgroups compared to their own main group reference sequence. Diagrams categorize frequencies following accessions; with identical sequences to the reference in grey, with at least one nucleotide substitution resulting in a synonymous variation in blue, with at least one amino acid variation (occurrence of presence > 5%) in orange and, with at least one rare amino acid variation (occurrence of presence < 5%) in green.

Overall data and results are illustrated and reported in Fig. 2 and Tables S2 and S3. We surveyed a total of 1397 accessions from the *Prunus* genus (Table S1). Sequences obtained from messenger RNA reverse transcription were allocated to three main groups following the subgenera in the *Prunus* taxomony: Armeniaca, Amygdalus and Persica (Fig. 1A). Subgroups include either (i) crop species (*P. armeniaca, P. dulcis, P. persica*), (ii) wild, undomesticated crop species (i.e. wild *P. armeniaca*) or (iii) wild related and ornamental species (species of the same group but distinct from the cultivated forms *P. armeniaca, P. dulcis* and *P. persica*). In this study, a haplotype corresponds to a set of linked/phased variants (alleles) located along the same chromosome and that are thus inherited together. Haplotypic richness was also calculated as the number of haplotypes per group or subgroup that differ from the reference haplotype (Tables S2 and S3).

At the *eIF4E* locus, all diversity parameters (substitutions/variations and heterozygosity) were the highest in the Amygdalus group compared to the Armeniaca and Persica groups (Fig. 2A, p-values < 0.0001 in Table S2B). Moreover, regardless of the main groups, wild related species showed a significantly higher haplotypic richness (*p*-values < 0.001) than crop species. Although data were not significantly different in few cases, the wild related species tend to display higher heterozygosity and a higher number of accessions with nucleotide substitutions and amino acid changes than the crop species (Table S2B).

At the *eIFiso4E* locus, the three main groups, i.e., Armeniaca, Amygdalus and Persica, showed significant differences for all parameters of diversity of eIFiso4E displayed in Table S3B (*p*-values < 0.0001). The Amygdalus group showed both the highest number of accessions with amino acid variation and haplotypic richness while the Armeniaca group had the highest number of accessions with heterozygosity and variations at the coding sequence level. The Persica group showed the lowest diversity for each criterion (Fig. 2B, Table S3B). Here also, wild related species for the three main groups showed significantly higher haplotypic richness than the crop species (*p*-values < 0.05, Table S3B). Moreover, as for *eIF4E*, wild related species showed a significantly higher heterozygosity level than the crop species, except for the Amygdalus group. While the frequency of accessions with variations in coding sequences compared to the reference accession is not significantly different between the three Armeniaca subgroups, amino acid variations are significantly higher in wild apricots (*p-*value < 0.0001, Table S3B). The most striking observation was for the Persica group in which wild related species showed a very significantly higher diversity than crop species (*p-*values < 0.0001 for all measured criteria in Table S3B).

### Genetic diversity of *eIF4E* and *eIFiso4E* among the three *Prunus* groups

Nucleotide (*π*) and haplotype (*Hd*) diversities were both calculated across the *eIF4E* and *eIFiso4E* gene sequences for the Armeniaca, Amygdalus and Persica groups (Table 2A). While *π* is used to measure the degree of polymorphism within a population or group of accessions, *Hd* is a measure of the uniqueness of a particular haplotype in a given population or a group of accessions.

**Table 2:** EIF4E and eIFiso4E genetic diversity parameters.

A	*π*		*Hd*		*dn/ds*		Tajima's *D*	
	*eIF4E*	*eIFiso4E*	*eIF4E*	*eIFiso4E*	*eIF4E*	*eIFiso4E*	*eIF4E*	*eIFiso4E*
Armeniaca group	0.00126	0.0029	0.3789	0.8016	0.27891	0.11826	− 2.28525	− 1.6838
Apricot crop species	0.00116	0.00279	0.3198	0.8293	0.55307	0.08791	− 2.28222**	− 1.24793
Wild apricots	0.00078	0.00201	0.3210	0.6117	0.09455	0.18278	− 1.71567	0.20376
Apricot related species	0.00286	0.00450	0.7417	0.8910	0.15252	0.07720	− 1.57057	− 1.28773
Amygdalus group	0.00503	0.00516	0.8160	0.9180	0.26722	0.15331	− 2.05692*	− 1.74178
Almond crop species	0.00255	0.00375	0.7308	0.8870	0.26898	0.13495	− 2.10693*	− 1.59196
Almond related species	0.00839	0.00634	0.9046	0.8537	0.32982	0.17519	− 1.56449	− 1.29035
Persica group	0.00142	0.00082	0.5752	0.1889	0.63500	0.03858	− 1.90355*	− 2.10157**
Peach crop species	0.00099	0.00022	0.5395	0.0438	2.45652	0.03191	− 1.83100*	− 1.97602*
Peach related species	0.00404	0.00313	0.7267	0.7401	0.16279	0.07125	− 0.77135	− 1.29487

B	*eIF4E*		*eIFiso4E*					
	Ratio of *π*	Ratio of *Hd*	Ratio of *π*	Ratio of *Hd*				
Amygdalus/Armeniaca	3.99206	2.15360	1.77931	1.14521				
Amygdalus/Persica	3.54225	1.41864	6.29268	4.85971				
Armeniaca/Persica	0.88732	0.65873	3.53659	4.24351				
Apricot related species/Apricot crop species	2.46552	2.31926	1.61290	1.07440				
Apricot related species/wild apricots	3.66667	2.31059	2.23881	1.45660				
Almond related species/Almond crop species	3.29020	1.23782	1.69067	0.96246				
Peach related species/Peach crop species	4.08081	1.34699	14.22727	16.89726				

A: Values of nucleotide diversity (*π*), haplotype diversity (*Hd*), ratio of substitution rate at non-synonymous to synonymous sites (*dn/ds*), and selection Tajima’s *D* statistics with corresponding significance values (* for *p* ≤ 0.05; ** for *p* ≤ 0.01, calculated with DNAsp v.5.10.01 software in the main groups and subgroups).

B: Ratios of *π*, *Hd*, and *dn/ds* between the main groups and between subgroups within the main groups.

For both genes,* π* values were globally in the same range (10^–3^) except for the wild apricots for *eIF4E* and for the peach crop species for *eIFiso4E* (10^–4^). Similar ranges of nucleotide diversity have already been observed in whole genome sequences of apricot and peach crop and wild species^26, 27^. In *eIFiso4E, Hd* was in the same range of values (10^–1^) as *eIF4E* except for the peach crop species subgroup (10^–2^). For both genes, *π* and *Hd* values were higher in all wild related species subgroups compared to the crop species ones, except, the *Hd* value for *eIFiso4E* in the almond crop species where it was slightly higher than that of the wild related species. Moreover, the Amygdalus group showed the highest values while the lowest ones were observed in the Persica group except for *Hd* of *eIF4E* in the Armeniaca group. Additionally, peach crop species displayed a significant drop in both *π* and *Hd* for *eIFiso4E* values in comparison with its wild related species (Table 2A,B).

The Tajima’s* D* statistics showed a negative value in all *Prunus* groups except for a slight neutral value at the *eIFiso4E* locus in wild apricots (e.g., 0.20376, Table 2A). The non-synonymous/synonymous substitution ratio, *dn/ds*, was found less than 1, showing evidence that synonymous mutations are more frequent than the non-synonymous ones, excepted in *eIF4E* for peach crop species (*dn/ds* = 2.45652, Table 2A). These ratio values also indicated that non-synomymous mutations were less prevalent on *eIFiso4E* compared to *eIF4E*, in particular in crop and wild related species of Armeniaca and Persica (*dn/ds*_*_eIFiso4E*_ < *dn/ds*_*_eIF4E*_, Table 2A). Moreover, at the whole gene level in all groups and subgroups, for both *eIF4E* and *eIFiso4E* the RELAX and BUSTED tests did not reveal any evidence of gene-wide relaxed nor intensified selection, neither positive or negative selection in the phylogeny of the genetic diversity of the sequences (*p-*values > 0.05).

### *eIF4E* and *eIFiso4E* haplotypes

At the amino acid sequence level, a total of 49 eIFiso4E haplotypes differing from the PPV susceptible haplotypes of the Armeniaca, Amygdalus and Persica groups were identified, whereas 99 haplotypes were counted for *eIF4E* (Table 3A). It was shown previously that PPV infection requires a functional eIFiso4F translation initiation complex^24, 25^. In consequence, we will hereafter focus exclusively on eIFiso4E haplotype variation. Susceptibility to the virus is predominant in the *Prunus* germplasm, therefore, susceptibility alleles of *eIFiso4E* are expected to be more common than the rare resistant alleles among the *Prunus* germplasm.

**Table 3:** Number of variable haplotypes and amino acid variations along the eIF4E and eIFiso4E sequences.

A								
Number of variable haplotypes:	Armeniaca		Amygdalus		Persica		3 *Prunus* groups	
	*eIF4E*	*eIFiso4E*	*eIF4E*	*eIFiso4E*	*eIF4E*	*eIFiso4E*	*eIF4E*	*eIFiso4E*
Differing from the susceptible reference haplotype	41	17	44	24	14	8	99	49
With occurrence < 5%	41	16	42	20	13	8	96	44
With occurrence < 5% and homozygous	12	8	12	10	8	5	32	23
With amino acid variation(s) inside the interaction domains	12/12 (6)	1/1 (1)	21/20 (6)	4/4 (3)	3/3 (3)	3/3 (2)		
With amino acid variation(s) inside interaction domain I	2/2 (0)	1/1 (**1**)	0	1/1 (0)	0	0		
With amino acid variation(s) inside interaction domain II	10/10 (**6**)	0	21/20 (6/**5**)	3/3 (**3**)	3/3 (**3**)	3/3 (**2**)		

B								
Number of:	Armeniaca		Amygdalus		Persica			
	*eIF4E*	*eIFiso4E*	*eIF4E*	*eIFiso4E*	*eIF4E*	*eIFiso4E*		
Positions with non synonymous variations along the amino acid sequence	32	19	32	20	16	8		
Amino acid variations	34	22	40	24	16	9		
Amino acid variations with occurrence < 5%	34	21	34	19	15	9		
Homozygous amino acid variations with occurrence < 5%	13	14	15	10	10	6		
Amino acid variations inside the interaction domains	4/4 (1)	2/2 (2)	1/0 (1)	3/3 (2)	1/1 (1)	1/1 (1)		
Amino acid variations inside interaction domain I	2/2 (0)	2/2 (**2**)	0	1/1 (0)	0	0		
Amino acid variations inside interaction domain II	2/2 (**1**)	0	1/0 (1)	2/2 (**2**)	1/1 (**1**)	1/1 (**1**)		

A: Variable haplotypes derived from the susceptible reference in the main groups regarding occurrence (< 5%), homozygosity and localization of amino acid variations inside the interaction domains (DI or DII) of the sequences. Numbers underlined, in brackets or in bold indicate the number of variable haplotypes with amino acid variations in the interaction domains respectively found with an occurrence < 5%, homozygous or homozygous with an occurrence < 5%

B: Number of positions with non synonymous variations and number of amino acid variations along eiF4E and eIFiso4E sequences regarding occurrence (< 5%), homozygosity, and localization of the amino acid variations in the interaction domains (DI or DII) of the sequences. Numbers underlined, in brackets or in bold indicate the number of amino acid variations respectively found with an occurrence < 5%, homozygous or homozygous with an occurrence < 5%.

Details of amino acid variations in haplotypes observed among the *Prunus* germplasm in the Armeniaca, Amygdalus and Persica groups are respectively depicted in Table S4A–C for *eIFiso4E*. As mentioned above, we did not comment data obtained for *eIF4E* (Table S5A–C). Indeed, we found that the *eIFiso4E* susceptible reference haplotypes are more prevalent than other haplotype frequencies in Persica (0.968085, Table S4C) and in Armeniaca (0.565189, Table S4A) as well as in Amygdalus (0.456054, Table S4B), if we overlook length polymorphism with the loss of one Alanine at position 28 (A28−) for *eIFiso4E*_Amy_P01.

Attention was first given to haplotypes found with a frequency lower than 5% in each main group (also called rare haplotypes) as reported in Table 3A. Rare haplotypes were identified both in the crop and the wild related species subgroups. eIFiso4E haplotypes differing from the susceptible reference were more often native of Asia (Central and Eastern Asia) and to the irano-caucasian region for Armeniaca (Table S4A) and Amygdalus with a Western European additional contribution for the latter (Table S4B). The few diverging Persica haplotypes came from Eastern Asia (Table S4C).

**Figure 3:**
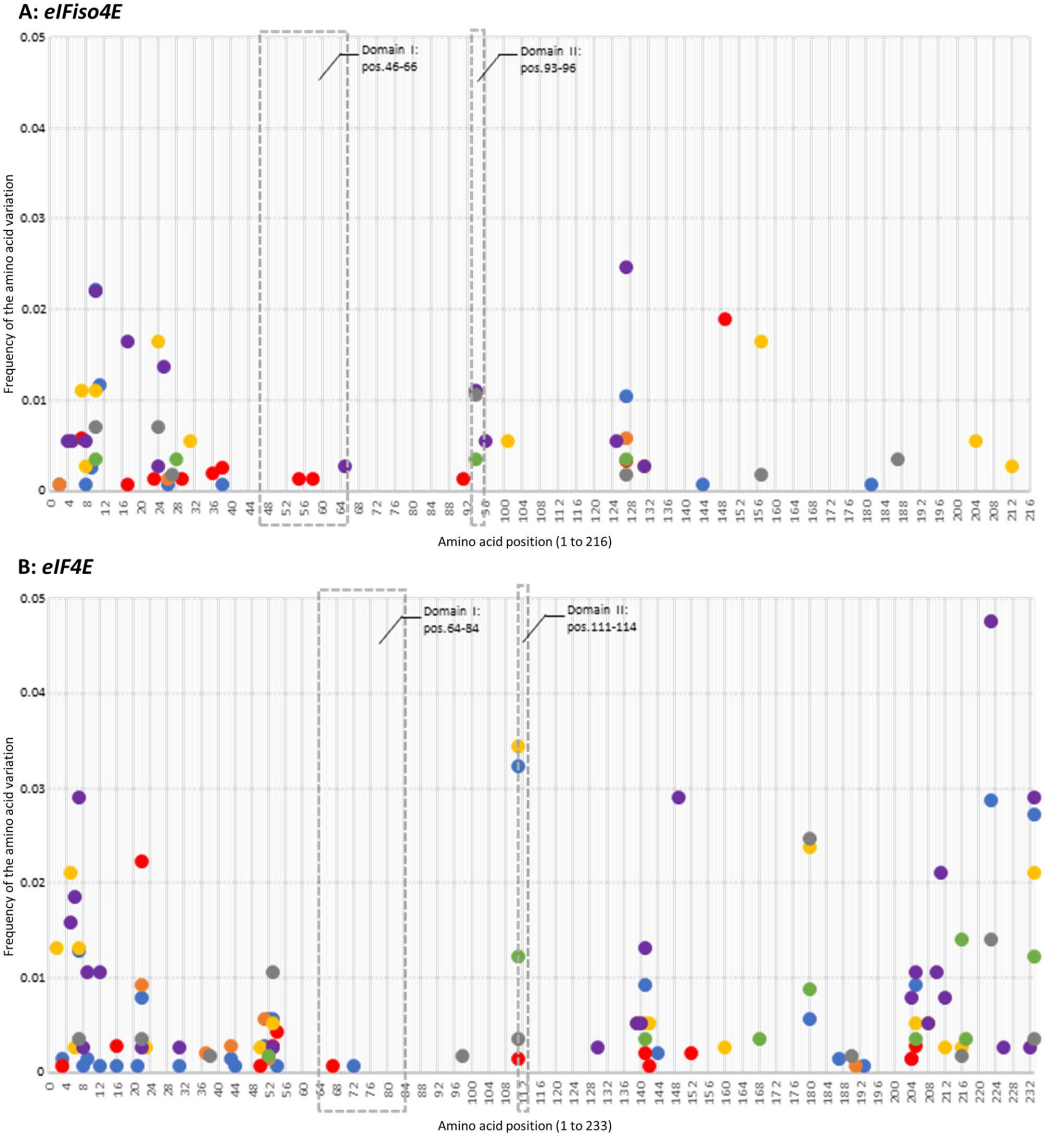
Frequency of rare amino acid variations (occurrence of presence < 5%) along eIFiso4E (**A**) and eIF4E (**B**) sequences. The interaction domains with the virus -Domain I and Domain II- are localized with their positions (pos.) on the sequences inside dotted grey rectangles. Each circle represents one amino acid variation retrieved at one specific position; each color refers to one subgroup as apricot crop species (blue), wild/undomesticated apricots (orange), apricot wild related species (red), almond crop species (yellow), almond wild related species (purple), peach crop species (green) and peach wild related species (grey).

Several positions along the amino acid sequences were identified with at least one non-synonymous variation compared to the chosen reference sequence; 19, 20 and 8 positions for Armeniaca, Amygdalus and Persica groups respectively (Table 3B). e*IFiso4E* is a susceptibility gene for which a homozygous mutation can lead to recessive resistance to PPV^20^, thus, we focused on homozygous variations over the *eIFiso4E* coding sequence. Among them, rare amino acid variations (occurrence < 5%) were selected under this threshold since resistance is less frequent than susceptibility in *Prunus* germplasm^16, 28^ (Table 3A,B). We further looked for variations within the two interaction domains, domain I (DI) and domain II (DII), involved in the plant/virus compatible interaction as described by^7^ (Tables S4 and S5 and Fig. 3A: *eIFiso4E*, Fig. 3B: *eIF4E*; data not commented). Along the eIFiso4E amino acid sequence, DI was delimited from position 46 to position 66 and DII from position 93 to position 96 (Fig. 3A). Additionally, we focused on variations impacting potentially the eIFiso4E 3D conformation and functionality that consequently may disturb the interaction with the viral protein but do not impact its role as a translation initiation factor. In the latter case, Meta-SNP, MutPred2 and PredictSNP online predictors were used to predict the impact of the variation(s) at the molecular and functional levels of the protein (Table S6A–C).

Regarding all the groups at the same time, one hot spots’ region of rare amino acid variations was identified along the eIFiso4E sequences, in the N-terminal region, within the first forty amino acids of the sequence whereby up to 90% of the non-synonymous rare variations mapped in this region (Fig. 3A, Table S4). Other variations were scattered along the amino acid sequence.

The analysis of the selection pressure for each site performed by the FUBAR test identified one amino acid variation per group that was positively selected (*p-*values < 0.05): D149E for Armeniaca, A127P for Amygdalus and N94S in DII for Persica.

In the Armeniaca group, the eIFiso4E_Arm_P15 haplotype was the only one described with homozygous amino acid variations in the interaction domain I at positions 55 and 58 (K55N and Q58K, respectively) (Table 3A). It was identified within the US_108 accession (cv. Yanmei, *P. mume*) originating from Japan (Tables 3A,B, S4A, S6A). Together with the K55N amino acid variation, four other variations at positions 2, 144 and 181 (A2E, A2V, Q144H and I181N) were predicted to have an effect in protein conformation. However, they were found in heterozygous allelic forms in the eIFiso4E_Arm_P04, eIFiso4E_Arm_P06, eIFiso4E_Arm_P09 and eIFiso4E_Arm_P17 haplotypes, respectively (Table S6A).

In the Amygdalus group, three different variations in the interaction domains were identified; S65T (DI) in the heterozygous eIFiso4E_Amy_P16, N94S (DII) in the homozygous eIFiso4E_Amy_P22 and eIFiso4E_Amy_P24, and D96E (DII) in the homozygous eIFiso4E_Amy_P21 with a potential effect on protein conformation for the latter (Tables 3A,B, S4B, S6B). Homozygous variations in DII were found in the almond related species *P. petunnikowii* (US_073 and US_189) for N94S *and P. tenella* (US_135) for D96E (Table S6B). Those three accessions came from Kazakhstan in central Asia (Table S1). Predictions of non-synonymous variations with a significant effect on protein conformation at other positions as R101G, T131I, T131P, K204I and R212L were all detected in heterozygous allelic forms (Tables S4B, S6B).

In the Persica group, four haplotypes were detected with the variation N94S in DII (Table S4C). This amino acid variation was found homozygous in eIFiso4E_Per_P01 and eIFiso4E_Per_P04 from the *P. persica* AZ_092 (Azerbaïjan) and the *P. davidiana* CH_002 (China) accessions, respectively. It was also found heterozygous in the eIFiso4E_Per_P04 and eIFiso4E_Per_P07 haplotypes (Table S6C). One single non-synonymous variation, E187D, that could result in a significant change in protein conformation was identified homozygous in the haplotype eIFiso4E_Per_P06 from the CH_154_3 accession (*P. davidiana*, China) (Tables S1, S6C).

The variation A127P was observed in all the three *Prunus* groups regarding the reference sequences (Fig. 3A) and was found homozygous in the Amygdalus *P. orientalis* TR_114, TR_115 (eIFiso4E_Amy_P05) and *P. webbii* US_193 accessions (eIFiso4E_Amy_P18). In Armeniaca, it was present in the homozygous eIFiso4E_Arm_P03 with *P. armeniaca* KZ_230_3 while in Persica, it was found heterozygous in the *P. davidiana* FR_AVI_056 accession (eIFiso4E_Per_P08).

Based on the above criteria i.e. non-synonymous and homozygous variation(s) potentially affecting the protein conformation of eIFiso4E and prioritizing the interaction domains, we selected a list of accessions to test for resistance to PPV under controlled conditions in a high confinement greenhouse (Table 4). Amino acid variations G38R in Armeniaca, A31P and S157N in Amygdalus were also targeted as they were homozygous and/or rare variations.

**Table 4:** List of selected accessions with eIFiso4E haplotypes derived from the reference sequence related to their main group.

Accession name	Main group	Sub-group	Species	*eIFiso4E* haplotype	Homozygous amino acid variation in the interaction domains	Homozygous amino acid variation in other positions	Phenotyping results
*KZ_230_3*	*Armeniaca*	*Wild apricots*	*P. armeniaca*	*eIFiso4E_Arm_P03*		***A127P***	Not tested
US_009	Armeniaca	Apricot related species	*P. brigantina*	eIFiso4E_Arm_P14		G38R	Highly susceptible
*US_108*	*Armeniaca*	*Apricot related species*	*P. mume*	*eIFiso4E_Arm_P15*	*DI: ****K55N****/****Q58K***	*L23V/A29T/S36I/F91L*	Not tested
AZ_203_5	Amygdalus	Almond related species	*P. fenzliana*	eIFiso4E_Amy_P07 & P12		A28-/S157N	No virus detected
AZ_205_4	Amygdalus	Almond related species	*P. fenzliana*	eIFiso4E_Amy_P07		A28-/A31P/S157N	No virus detected
AZ_210_1	Amygdalus	Almond related species	*P. fenzliana*	eIFiso4E_Amy_P07		A28-/A31P/S157N	No virus detected
AZ_210_2	Amygdalus	Almond related species	*P. fenzliana*	eIFiso4E_Amy_P01 & P07		A28-/S157N	No virus detected
AZ_215_1	Amygdalus	Almond related species	*P. fenzliana*	eIFiso4E_Amy_P07		A28-/A31P/S157N	No virus detected
AZ_219_1	Amygdalus	Almond related species	*P. fenzliana*	eIFiso4E_Amy_P01 & P07		A28-/S157N	Moderately susceptible
KR_019_6	Amygdalus	Almond crop species	*P. dulcis*	eIFiso4E_Amy_P05		A28-/**A127P**	Weakly susceptible
*TR_114*	*Amygdalus*	*Almond related species*	*P. orientalis*	*eIFiso4E_Amy_P18*		*A17V/A28-/****A127P***	Not tested
*TR_115*	*Amygdalus*	*Almond related species*	*P. orientalis*	*eIFiso4E_Amy_P18*		*A17V/A28-/****A127P***	Not tested
US_012	Amygdalus	Almond crop species	*P. dulcis*	eIFiso4E_Amy_P12		A28-/S157N	Weakly susceptible
US_037	Amygdalus	Almond crop species	*P. dulcis*	eIFiso4E_Amy_P01 & P12		A28-/S157N	No virus detected
*US_073*	*Amygdalus*	*Almond related species*	*P. petunnikowii*	*eIFiso4E_Amy_P24*	*DII: ****N94S***	*A28-*	Not tested
US_135	Amygdalus	Almond related species	*P. tenella*	eIFiso4E_Amy_P21	DII: **D96E**	V10-/A28-	No virus detected
*US_189*	*Amygdalus*	*Almond related species*	*P. petunnikowii*	*eIFiso4E_Amy_P22*	*DII: ****N94S***	*V5L/A8T/V10-/A28-*	Not tested
US_193	Amygdalus	Almond related species	*P. webbii*	eIFiso4E_Amy_P18		A17V/A28-/**A127P**	Weakly susceptible
*AZ_092*	*Persica*	*Peach crop species*	*P. persica*	*eIFiso4E_Per_P01*	*DII: ****N94S***	*V10-/A127G*	Not tested
*CH_002*	*Persica*	*Peach related species*	*P. davidiana*	*eIFiso4E_Per_P04*	*DII: ****N94S***	*E24D*	Not tested
CH_154_3	Persica	Peach related species	*P. davidiana*	eIFiso4E_Per_P06		**E187D**	No virus detected

Haplotypes were selected following several criteria (i) a homozygous amino acid variation as a mandatory criterion, (ii) an amino acid variation in at least one interaction domain and/or with a significant predicted effect (see Table S6), (iii) a rare amino acid variation (occurrence < 5%) and (iv) accessions with budsticks available to be grafted. Accessions in italics were not phenotyped due to unsuccessful grafting. Amino acid variations in bold were predicted to have a significant effect on protein conformation/function. Other amino acid variations targeted in this study are underlined. The symbol "-" represents an amino acid deletion.

**Figure 4:**
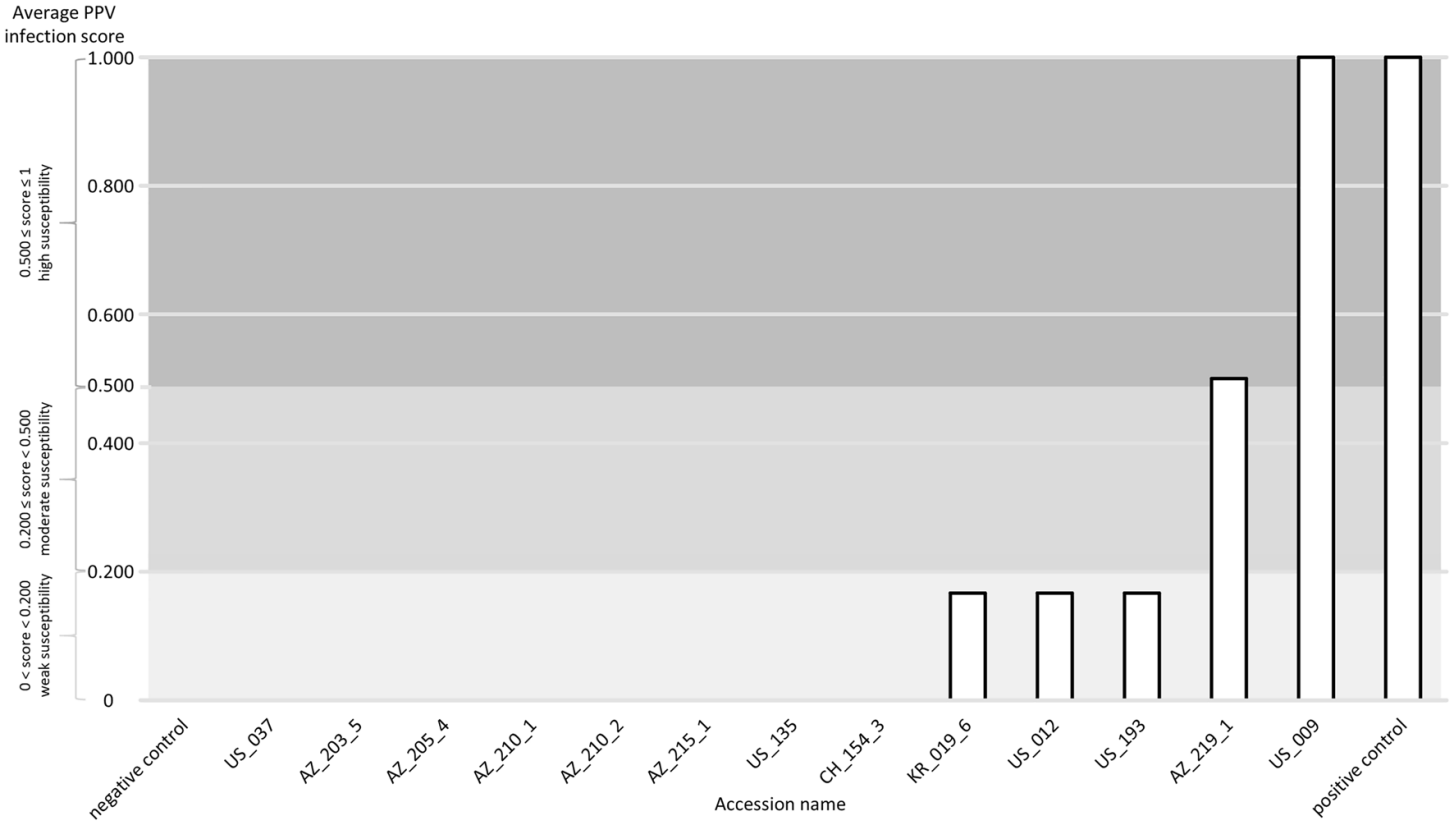
Average PPV infection score after successive cycles of phenotypic evaluation for the selected accessions (see Table 4). Resistance to PPV and the negative control are characterized by a null score (0). Three levels of susceptibility are categorized from a weak (0 < score < 0.200), then a moderate (0.200 ≤ score < 0.500) to a high susceptibility score (0.500 ≤ score ≤ 1). The positive control is set to 1.

In total, 13 accessions were phenotyped up to three successive vegetative cycles (Table 4). The presence/absence of viral particles was estimated by serological assays (ELISA) using a PPV-specific antibody. No virus was detected over three cycles for seven Amygdalus accessions: one almond crop species *P. dulcis* US_037 and six almond wild related species among which five representatives of *P. fenzliana* (AZ_203_5, AZ_205_4, AZ_210_1, AZ_210_2, AZ_215_1) and one *P. tenella* (US_135). One representative of the peach wild related species, *P. davidiana* CH_154_3 was also scored PPV negative (Fig. 4). Intermediate susceptibility levels were measured in Amygdalus *for P. dulcis* KR_019_6 and US_012 as well as in *P. webbii* US_193. Amygdalus *P. fenzliana* AZ_219_1 and Armeniaca *P. brigantina* US_009 were moderately to highly susceptible, respectively (Table 4, Fig. 4, Table S7). In total, seven accessions were resistant to sharka. They will be further studied in progenies from crosses of these resistant accessions with PPV susceptible accessions, to verify co-segregation between the newly identified allelic variations in *eIFiso4E* and resistance to sharka.

## Discussion

Because of their small genome size (a few kilobase pairs), viruses require (thus highjack) host proteins to complete their entire infectious cycle in the host plant, from viral RNA translation to virus movement, virion assembly and disassembly and viral replication^29^. In the case of PPV, two previous studies demonstrated the central role of components from the translation initiation complex, eIFiso4F, in the PPV-compatible infection of stone fruit species (*Prunus* spp.)^24, 25^. Previous studies identified resistance to PPV in *P. davidiana*, in crop almond (*P. dulcis*) and in crop apricot (*P. armeniaca*) through quantitative trait phenotyping^16, 28, 30, 31^. One major locus controlling resistance to sharka in apricot has been mapped on linkage group 1 and named PPVres^18, 32, 33^. While the *eIFiso4E* gene maps on the upper end of the chromosome 1^30^, it does not co-localize with PPVres. Therefore, finding eIFiso4E allelic variations impaired in Prunus-PPV interactions would provide new sources of resistance to sharka that could be combined with previously identified ones.

In the current study, the genetic diversity in the coding sequences of the recessive resistance gene *eIFiso4E* and of its counterpart, *eIF4E*, was analyzed in three *Prunus* crop species (apricots, almonds and peaches) and their wild related species from eleven geographic regions. The aim was to infer the evolutionary pattern of both genes involved in cellular translation initiation, to identify natural allele variations from susceptibility alleles and to evaluate the potential impact of these variations on *Prunus*/PPV interactions.

Polymorphism at both genes showed that the genetic diversity was globally higher among the wild related species than apricot, almond and peach crop species. Despite nucleotide variability presents in the 1,397 accessions screened here, none of them displayed null alleles due to frameshift variations causing truncated proteins. Low proportions of non-synonymous variations (*dn/ds* < 1 in the three main groups) likely indicated the removal of mutations that could be potentially deleterious or that could impair the proper functioning of the translation initiation complex. Likewise, Tajima’s *D* statistics for the both loci showed negative values, thus demonstrating an absence of neutrality between the mean pairwise difference and the number of segregating sites, explained by an abundance of low frequency haplotypes and the *dn/ds* ratios. Interestingly, the eIFiso4E coding sequence appeared more constrained than *eIF4E* with a *dn/ds* ratio consistently lower for *eIFiso4E* than for *eIF4E,* except for the wild undomesticated apricots. That would indicate a higher cost of non-synonymous mutations at the *eIFiso4E* locus than *eIF4E*. This result is consistent with the hypothesis that non-functional or silenced *eIFiso4E* alleles are lethal or deleterious in diploid *Prunus* growth^25^. Fitness cost of loss-of-function in *eIFiso4E* has been previously suspected for melon^34^. The situation is different in wild apricots because of their self-incompatibility and thus an expected higher rate of heterozygosity would allow the occurrence of a higher number of non-synonymous mutations at the *eIFiso4E* locus.

Regarding the number of accessions with amino acid variations compared to those from the susceptible accessions, the estimators for: the haplotypic richness, the level of heterozygosity, the haplotype diversity (*Hd*) and the nucleotide diversity (*π*) were all significant strong indicators that *Prunus* related species actually constitute a reservoir of genetic diversity for alleles of interest. Three recent studies confirmed this view. In 2019, two consecutive studies showed that nucleotide diversity in peach wild related species was twice as much as that of peach landraces or peach improved cultivars^26, 35^. More recently in 2021, Groppi et al.^27^ showed that undomesticated (wild) apricots from central Asia had global nucleotide diversity 1.5 times higher than cultivated European domesticated apricots.

When comparing genetic diversity within the *Prunoïdeae* subfamily, we observed that eIF4E genetic diversity was the highest for the Amygdalus group while that of the *eIFiso4E* locus was highest for the Armeniaca group. In contrast to these two loci, members of the Persica group displayed the lowest value of genetic diversity (*π*), the lowest frequency of amino acid variations and the lowest level of heterozygosity. Consequently, the identification of potential variations in candidate genes is more likely to be successful firstly in related species and secondly in Amygdalus and Armeniaca than in Persica. Those accessions we found exhibiting allelic variations that potentially affected the overall protein conformation or the plant-potyvirus interaction domain(s) were all tested for susceptibility to sharka and eight out of thirteen were resistant to PPV, among which six were from the almond wild related species, *P. fenzliana* (5) and *P. tenella* (1)*,* and one was from a peach related species, *P. davidiana*. Only one of them were from crop species (almond, *P. dulcis*).

Provided that the observed resistance is indeed genetically controlled by the corresponding variations in the eIFiso4E coding sequence, our data provide new PPV resistant genitors. Regarding the amino acid variations; A28- and S157N are present in both susceptible (AZ_219_1, US_012, US_193) and resistant (AZ_203_5, AZ205_4, AZ210_1, AZ_210_2, AZ_215_1, US_037) accessions, thus, they are not correlated with the observed resistance phenotype. Nevertheless, further analyzes need to be performed for the amino acid variations V10- (US_135), A31P (AZ_205_4, AZ_210_1, AZ_215_1), D96E (US_135) and E187D (CH_154_3), all associated with PPV resistant accessions. Co-segregation between the above non-synonymous variations and response to PPV infection will have to be tested in F2 progenies because of the recessive nature of the resistance trait. Association between eIF4E variation and PPV infection was not tested here because previous studies demonstrated a role of the eIFiso4F complex in PPV infection but not for the eIF4F complex^24, 25^. However, those results were based on the infection of eIFiso4E- or eIFiso4G-silenced plum by the M and D PPV strains. This does not preclude a potential role of the eIF4F complex into the infection of cherry trees by more distinct strains such as PPV-C, thus providing further prospects to our eIF4E diversity sequence data.

Such investigation of genetic diversity in natural populations for one or several genes, also called EcoTILLING was developed as a non-transgenic reverse genetic approach in animals and plants to screen the natural diversity of targeted genes in many species^36^. This strategy was successfully used to identify novel alleles for candidate genes involved in the resistance to diseases and specifically for the eIF susceptibility genes in melon^10^, pepper^8^ and barley^37^. Our study reports the first screening of genetic diversity of two eIF genes in perennial fruit trees, i.e. *Prunus* species including both the crop and the wild (undomesticated and related species) germplasm. Novel *eIFiso4E* alleles in several *Prunus* species that could be associated with the resistance to PPV were identified; it provides initial insights on functional, genetic diversity and potential new sources of resistance to sharka.

## Materials and methods

### Plant material and sampling

The study includes a total of 1397 accessions from the *Prunus* genus (Table S1). Samples were allocated to three main groups following the subgenera in the *Prunus* taxomony (Fig. 1A). The first group corresponds to representatives of the Armeniaca group (n_Armeniaca_ = 892) divided into three subgroups representing (i) apricot crop species (*Prunus armeniaca*) (n_apricot_crop_species_ = 475), (ii) wild, undomesticated apricots (*P. armeniaca*) (n_wild_apricots_ = 315) and (iii) apricot wild related and ornamental species (n_apricots_related_species_ = 102). The second main group was Amygdalus (n_Amygdalus_ = 210) divided between (i) almond crop species (*P. dulcis*) (n_almond_crop_species_ = 138) and (ii) almond wild related species (n_almonds_related_species_ = 72). The last main group was Persica (n_Persica_ = 295) composed of (i) peach crop species (*P. persica*) (n_peach_crop_species_ = 260) and (ii) peach related species (n_peach_related_species_ = 35). We acknowledge the highly valuable contribution of local collaborators and curators of the National repositories who undertook the formal identification of the plant material used in this study: M. Delmas assisted by J-M Audergon and H. Duval for the plant material held at the French GRC, J. Preece for the material issued by the ARS-USDA, B. Krska for the Czech Horticultural repository, B. M. Asma for the Turkish germplasm, G. Balakishiyeva and A. Mammadov for the Prunus species growing in Caucasia, T. Turdiev and T. Kostritsyna assisted by the late R. Karychev and W. Liu for the Chinese germplasm.

Apricots, almonds and peaches are crop species that comprise modern varieties, breeding genitors, ancient local varieties or landraces. Wild, undomesticated apricots correspond to accessions sampled away from the cultivated areas in the natural forest mountains of Central Asia. Wild related and ornamental species differ phylogenetically from the *P. armeniaca*, *P. dulcis* and *P. persica* crop species and were considered as related species of apricots, almonds and peaches, respectively. Accessions were sampled in different geographic areas; in orchards and different germplasm repositories, in private gardens, along the roads, or in natural forest mountains (Fig. 1B). For full details, see Table S1.

### RNA extraction

Around 0.1 to 0.3 mg of fresh or lyophilized young leaves were collected in a 2 ml tube with two iron balls and then stored at − 80 °C. Samples were ground to obtain a uniform powder. Total RNAs were immediately extracted with the Macherey–Nagel NucleoSpin RNA plant extraction kit (http://www.mn-net.com). To increase the yield of total RNAs, the extraction procedure was modified by the addition of 1% of beta-mercaptoethanol and 1% w/v of PVP40 in the RAP extraction buffer. Only total RNAs with an absorbance ratio (A_260/280 nm_) of 2 to 2.2 were used to pursue the analysis, otherwise RNAs were re-extracted. RNAs were then stored at − 80 °C.

### Reverse transcription and gene amplification by PCR

cDNAs were synthesized from total RNAs in 96-well plates with the RevertAid H Minus Reverse Transcriptase and the oligo(dT)_18_ primer according to the manufacturer’s protocol (http://www.thermoscientific.com).

Two unique couples of specific primers for the amplification of the full-length open reading frames of the candidate genes were designed according to the published peach genome sequence in the Genomic Database for Rosaceae (GDR, http://www.rosaceae.org) and were used without primer sequence modification to perform all the polymerase chain reactions (PCRs) in the peach, almond, apricot and their related species genomes. The candidate genes are referenced as Prupe.4G072600.1 for *eIF4E* (coding sequence length: 705 bp), and Prupe.1G046600.1 for *eIFiso4E* gene (645 bp). For both genes and each accession, PCRs were performed in a final volume of 25 µL using the Taq DNA Polymerase from Qiagen (http://www.qiagen.com) with a modified mix proportions as followed; 10X Buffer/15 mM MgCl2 (1X final), 25 mM MgCl_2_ (to reach 2.5 mM final), dNTPs mix (0.25 mM final each), forward and reverse primers (0.5 µM final each), 5 U/µl Taq Qiagen (0.625 U per reaction) and 2 µl of cDNAs matrix (20–50 ng/µl). PCR cycling conditions were 3 min at 94 °C for general denaturation followed by a one 3-step cycle repeated 40 times (denaturation for 30 s at 94 °C, gene-specific primers’ annealing temperature for 30 s and extension 40 s at 72 °C) and a final extension for 10 min at 72 °C. To perform PCR for *eIF4E*, primers’ annealing temperature was optimized at 61 °C with the forward primer 5′-CGCCAAGAAAGAAAAGCGAG-3′ and the reverse primer 5′-GCAAAGAACAATATACACATCA-3′ and for *eIFiso4E* the annealing step was performed at 58 °C with the forward primer 5′-AAACAACACAACCCCGACAG-3′ and the reverse primer 5′-TCAAACATTGTATCGA-3′. PCR products were verified by electrophoresis with a 1.5% agarose gel and visually quantified by comparison with the MassRuler DNA ladder from ThermoScientific.

### Allelic sequencing, phasing and alignment

PCR products were sequenced with the Sanger method by the Genewiz company, following recommendations available on the http://www.genewiz.com website. Quality of sequences was verified from the chromatograms using Chromas v.2.5.1 (https://technelysium.com.au/wp/chromas/). Sequences of accessions were all classified in a data file corresponding to the appropriate main group and subgroup in which they belonged (see “Plant material and sampling” section). As the *Prunus* species used in the current study are all diploid, each gene can have up to two alleles. In this case, heterozygous allelic forms with unphased genotypic data were rebuilt with the ELB algorithm method implemented in Arlequin v.3.5.2.2 as a pseudo-Bayesian approach to specifically estimate gametic phase in recombining sequences^38^. Sets of phased alleles also called haplotypes were first aligned altogether using ClustalW Multiple Alignment method (1000 Bootstraps) using BioEdit v.7.1.3.0 software^39^, then trimmed to deal with nothing else than the coding sequence from the start to the stop codons and finally translated into amino acid sequences.

### Polymorphism detection and statistical analyses

For each main group and subgroup, amino acid sequences were compared to a reference haplotype corresponding to the sequence coming from a susceptible accession to PPV i.e., the apricot cultivar ‘Moniqui’ (*P. armeniaca*), the almond cultivar ‘Aï’ (*P. dulcis*) and the peach rootstock ‘GF305’ (*P. persica*) for the Armeniaca, Amygdalus and Persica groups and subgroups respectively (see “Plant material and sampling” section). These sequences of reference were respectively called Arm_P00, Amy_P00 and Per_P00 (“P” for protein) with the prefix “eIF4E_” or “eIFiso4E_” (i.e. eIF4E_Arm_P00). For each new haplotype detected, the number after “P” was incremented (“P01”, “P02”, …).

For both coding and amino acid sequences levels of homozygosity, heterozygosity, haplotypic richness and the presence of variations were evaluated and compared between groups and subgroups using Chi-square tests (*χ*^2^ tests) statistical analyses performed by XLStat software v.2020.1.3. Frequencies of haplotypes and amino acid variations were also calculated and were called rare haplotypes and rare amino acid variations when their occurrences were less than 5%.

### Genetic diversity parameters

Genetic diversity estimates were calculated using DNAsp v.5.10.01 software^40^. The haplotype diversity (*Hd*), the nucleotide diversity (*π*), the ratio of non-synonymous to synonymous substitutions (*dn/ds* ratio) and the Tajima’s *D* statistic were calculated from allelic data for each main group and subgroup. The Datamonkey online database (http://www.datamonkey.org) was used to test the selection pressure on (i) the whole gene sequences with the RELAX test to analyze whether the strength of the selection has been relaxed or intensified along the phylogeny of the sequences and the BUSTED model (Branch-site Unrestricted Statistical Test for Episodic Diversification) to provide a gene-wide test for positive selection at at least one site on at least one phylogenetic branch, and (ii) at sequence sites with the FUBAR test (Fast Unconstrained Bayesian AppRoximation) for large data sets with a Bayesian approach to infer the *dn/ds* ratio on a per-site basis and detect positive or negative pervasive selection at the amino acid level assuming the selection pressure for each site is constant along the entire phylogeny^41^.

### Predicting effects of amino acid variations on protein conformation/function

To predict the potential effect of amino acid variations on the protein conformation and/or function, three computational methods were used: (i) Meta-SNP (https://snps.biofold.org/meta-snp/index.html) a random forest-based binary classifier predictor combining predictions of four methods (SNAP2, SIFT, PANTHER, PhD-SNP) and four elements extracted from the PhD-SNP protein sequence profile based on training dataset derived from SwissVar^42^, (ii) MutPred2 (http://mutpred.mutdb.org) an algorithm able to quantify the pathogenicity of amino acid substitutions and describe how they can affect the protein function by modeling a broad repertoire of structural and functional alterations from amino acid sequence^43^ and (iii) PredictSNP (https://loschmidt.chemi.muni.cz/predictsnp1/) a consensus classifier combining eight prediction methods (MAPP, PhD-SNP, PolyPhen-1/-2, SIFT, SNAP, nsSNPAnalyser, PANTHER) to provide a more accurate and robust alternative to the predictions based on accession integrated tools and weighted by the method-specific confidence scores^44^. Even though these softwares based their predictions on mammal (mostly humans) databases (no such plant-specific predictors exist), they enabled the classification of amino acid variations found along the eIFiso4E protein for Armeniaca, Amygdalus and Persica accessions to select variable haplotypes for testing PPV infection in the greenhouse.

### Phenotypic evaluation

Once the selection of accessions with variable haplotypes was established, phenotypic evaluation of PPV resistance was performed in a high confinement greenhouse following the protocol described in^14^. We used the same PPV Marcus isolate (PPV M20), maintained on GF305 indicator seedlings, for all tests because it is infecting equally and successfully accessions of the Armeniaca, Amygdalus and Persica groups, which is not the case of the other strains (D Dideron, C Cherry etc.…)^18^. Indeed, although PPV M and PPV D are the most common and among them, PPV-D is considered as the most epidemiologically competitive and the most widespread worldwide, PPV-D isolates are less efficiently transmitted than PPV M in almond and peach^13^. Three technical replicates per accession were first inoculated with PPV M20 and then scored over three consecutive, vegetative cycles of observations with two rounds of measurements each by serological assays (ELISA). Technical replicates consist in the same accession grafted on three independent PPV-susceptible rootstocks. One vegetative cycle consists in a succession of 3 months of dormancy in a cold chamber followed by 3 months of growth in the greenhouse. To look for the presence of the virus in the leaves, viral particles were quantified by ELISA, providing optical density values (OD). When OD was at least twice higher than the OD value of the negative control (the non-infected cultivar GF305), the sample was considered as infected. In this case, a score of 1 was attributed to the sample. Otherwise, the score was 0. After each cycle, the average response score was calculated from the two ELISA measurements while at the end of the phenotyping tests, the global average score was obtained by averaging data from the three complete cycles. Accessions were considered resistant with a global score of 0. Three levels of susceptibility were then categorized with a weak (0 < score < 0.200), a moderate (0.200 ≤ score < 0.500) and a high (0.500 ≤ score ≤ 1) score.

### Statements

Most of the samples used in this study were collected before October 2014, in the frame of the FP7 IRSES-246795 “STONE” project. Appropriate permissions from responsible authorities for collecting and using *Prunus* samples from Central Asia, Caucasia and China were obtained by the local collaborators. The rest of the samples were kindly provided, with due authorizations, by the curators of the French INRAE Genetic Resources Centre (GRC, Bourran), the US ARS-USDA repository, the Lednice germplasm collection; further details are available on their respective databases.

### Supplementary Information

Supplementary Tables.

## Data Availability

All the raw sequencing data generated during the current study were deposited in the SRA under project number ID: PRJNA918999. No voucher specimen was deposited in publicly available herbarium, however live samples are available through the National repositories: French Genetic Resources Centre, US ARS-USDA repository and Czech Horticultural repository of Lednice.
